# Brexpiprazole in the treatment of behavioral symptoms of dementia: a case report

**DOI:** 10.1192/j.eurpsy.2025.1737

**Published:** 2025-08-26

**Authors:** A. Izquierdo De La Puente, P. Del Sol Calderon, R. Fernandez Fernandez

**Affiliations:** 1 Psiquiatria, Hospital Universitario Puerta de Hierro de Majadahonda; 2 Psiquiatria, Hospital Universitario Infanta Cristina, Madrid, Spain

## Abstract

**Introduction:**

A case report on the use of Brexpiprazole for the treatment of behavioral disturbance in dementia is presented.

**Objectives:**

A brief review of the benefit of Brexpiprazole treatment in the treatment of dementia is presented in a case report.

**Methods:**

This is an 84-year-old female patient with newly diagnosed multifactorial cognitive impairment. The patient was brought to Mental Health because of the behavioral alteration presented. She reported delusions of harm, theft and a suspicious attitude in relation to moderate cognitive impairment, as well as the recent transfer to a nursing home. In addition, the nursing home had observed that he presented erratic wandering that affected the functioning of the residential environment. In response to this symptomatology, the patient was aggressive and physically heteroagressive towards the caregivers.

The patient, due to the clinical presentation, had been treated with benzodiazepines, which had worsened the episodes of agitation and confusion, interspersed with episodes of somnolence. Therefore, her treatment was modified by adding quetiapine and haloperidol, worsening her psychomotor restlessness and alertness.

**Results:**

When the patient was seen in the psychiatry department, she presented a high level of restlessness that corresponded to akathisia due to the haloperidol, as well as a fluctuating level of alertness that oscillated between wakefulness and somnolence. Despite the overmedication, according to the residency report, the patient maintained episodes of agitation and heteragresivity during wakefulness.

For this reason, it was decided to replace the antipsychotic treatment of quetiapine and haloperidol, progressively with brexpiprazole at 4mg DMD divided in two.

After two weeks of monotherapy with brexpiprazole, the side effects of the previous treatment disappeared, and the patient’s daily functioning improved. She remained alert, the suspicious attitude and the delusions of harm disappeared. The episodes of behavioral disturbances had also ceased.

**Conclusions:**

For the treatment of behavioral symptoms in dementia, it is important to have an effective approach to the clinical management without causing adverse effects that can be severe in elderly people. Brexpiprazole is an atypical antipsychotic, being a 5HT1A and D2 partial agonist and a 5HT2A antagonist, and is an appropriate treatment in this age group.

**Disclosure of Interest:**

None Declared

